# Buffalo Dental College

**Published:** 1887-04

**Authors:** 


BUFFALO DENTAL COLLEGE.—Several eminent dentists in Buffalo have been granted permission by the trustees of the Niagara University to organize a College of Dental Surgery. A full notice will appear in the JOURNAL next month.

